# Overt orienting of spatial attention and corticospinal excitability during action observation are unrelated

**DOI:** 10.1371/journal.pone.0173114

**Published:** 2017-03-20

**Authors:** Sonia Betti, Umberto Castiello, Silvia Guerra, Luisa Sartori

**Affiliations:** 1 Dipartimento di Psicologia Generale, Università di Padova, Padova, Italy; 2 Center for Cognitive Neuroscience, Università di Padova, Padova, Italy; 3 Centro Beniamino Segre, Accademia Nazionale dei Lincei, Roma, Italy; University of Bologna, ITALY

## Abstract

Observing moving body parts can automatically activate topographically corresponding motor representations in the primary motor cortex (M1), the so-called *direct matching*. Novel neurophysiological findings from social contexts are nonetheless proving that this process is not automatic as previously thought. The motor system can flexibly shift from imitative to incongruent motor preparation, when requested by a social gesture. In the present study we aim to bring an increase in the literature by assessing whether and how diverting overt spatial attention might affect motor preparation in contexts requiring interactive responses from the onlooker. Experiment 1 shows that overt attention—although anchored to an observed biological movement—can be captured by a target object as soon as a social request for it becomes evident. Experiment 2 reveals that the appearance of a short-lasting red dot in the contralateral space can divert attention from the target, but not from the biological movement. Nevertheless, transcranial magnetic stimulation (TMS) over M1 combined with electromyography (EMG) recordings (Experiment 3) indicates that attentional interference reduces corticospinal excitability related to the observed movement, but not motor preparation for a complementary action on the target. This work provides evidence that social motor preparation is impermeable to attentional interference and that a double dissociation is present between overt orienting of spatial attention and neurophysiological markers of action observation.

## Introduction

Direct evidence in favor of a functional continuum between action-observation and action-execution (direct matching) has been reliably produced using transcranial magnetic stimulation (TMS) over the primary motor cortex (M1): passive observation of human actions results in a correspondent modulation of the observer’s corticospinal excitability (CE) [1]. The first indication that CS excitability is modulated not only during voluntary movements, but also during action observation was provided by Fadiga and colleagues in 1995 [2]. Since then, some research groups have been able to replicate these findings and other experiments have been designed [3–5]. In 2001 Gangitano and colleagues demonstrated that the execution-observation matching system is linked to the observed action even in terms of its temporal coding [6]. Along these lines, Urgesi and colleagues [7,8] found that direct matching is maximal for snapshots evoking ongoing but incomplete actions. The link between action perception and corresponding motor activation has been recently ascertained with causal methods, beyond pure correlational evidence (for review, see [9]). For example, interfering with premotor regions of the execution-observation matching system was found to impair action recognition [10–13]. Interestingly, studies have also found that TMS conditioning of the same premotor regions alters the modulation of CS excitability during action observation [14–18]. Overall, these results provide compelling evidence that the frontal component of the observation-execution matching system plays an important role in the coding of others' motor behaviors. The underlying mechanism of direct matching is thought to be the automatic activation of motor representations of topographically similar actions to those being observed (for review, see [19]). To date, whether or not this visuomotor transformation process is automatic is currently under debate [20,21].

The traditional distinction between automatic and controlled processes is that the former are triggered involuntarily and do not require attention to select relevant visual information [22]. In this perspective, if observing an action automatically triggers an increase in CE, then a perceived action should be processed even in the absence/reduction of attentional resources [23]. To the extent that attention is critical for direct matching to occur, instead, CE should diminish whenever a participant's attention is diverted from an observed movement. Support to the latter comes from behavioral [24,25], neuroimaging [26–28] and neurophysiological [29,30] findings. Evidence suggests that participants are faster to initiate a hand movement that is congruent with a concurrently observed action, relative to one that is incongruent, and that this process is susceptible to top-down modulations. In particular, spatial attention has to be directed towards a body part for effector compatibility effects to emerge [24,31]. Interestingly, imitative compatibility effects no longer occur when participants direct their attention away from the movement-relevant features of the stimulus, thus highlighting the crucial role of selective spatial attention [25]. Moreover, specifically attending to our own performed actions can reduce the motor interference produced by the observation of other’s actions (i.e., actions observed from an allocentric perspective; [32]), suggesting that top-down factors may influence the direct matching.

In neural terms, Chong and colleagues [26] used functional magnetic resonance imaging (fMRI) to determine whether cortical activity associated with action observation is modulated by the strategic allocation of selective attention. Participants performed a low attentional load or high attentional load visual discrimination task while observing a reach-to-grasp hand action. The activity within the action observation network was not altered by attentional load. The automatic activation of these areas may represent the neural bases of behavioral findings that reaction time increases when participants perform actions that are incongruent with those they observe [33,34]. Crucially, however, a specific reduction of left inferior frontal gyrus (IFG) activity under conditions of high attentional load for biological movement seems to reflect our ability to selectively filter task irrelevant actions during ongoing behavior [26]. Also magnetoencephalic (MEG) response to point-light biological motion displays, although largely independent of attention, seems to reflect further processing when stimuli are attended [28]. In neurophysiological terms, Leonetti and colleagues [35] asked participants to gaze upon a fixation point while covertly attending to an action sequence, in order to test whether presenting an action in peripheral vision could differently modulate motor excitability. The results showed that, even if the action viewed in peripheral vision—and then covertly attended—was effective in modulating the excitability of motor pathways, the accuracy of the motor response was low and rough. Along this line, other researchers adopted subliminal presentation of implied action images, demonstrating that the perceptual awareness of the action stimuli is required for motor resonance to occur ([36]; see also [37] for a behavioral demonstration). Schuch and colleagues [30] in an EEG study investigated the mu rhythm (oscillatory activity over sensorimotor cortex) and reported stronger activations of the motor system—as revealed by mu rhythm suppression—when an observed grasping action was relevant to the observers’ task (i.e., when they were later judging the grasp than when judging a colour change). Taken together, these data seem to suggest that motor system activation during action observation can be automatic, but attentional filters are at play to limit unnecessary processing and mimicry of observed actions [but see [23] for alternative hypothesis].

The question of whether attention is required for direct matching to occur also addresses the debate regarding the presence of special classes of stimuli with particular biological and social significance, which require less attentional resources to be processed [38,39]. Therefore, the adoption of social stimuli such as body movements requiring the involvement of the observer can provide a valid testing ground to investigate the role of spatial attention during social interactions.

Here we capitalized on a visuo-motor paradigm, which has the ability to modulate an onlooker's CE during observation of complementary requests [40]. Complementary actions are defined as “any form of social interaction wherein two or more individuals coordinate and mutually complete their incongruent actions, rather than performing imitative behaviors” [41]. If someone holding a mug by its handle hands it to us, we automatically select the right grip to take it. In this case, the two grips adopted by the two individuals are complementary (mismatched). Notably, this visuo-motor paradigm allows revealing spontaneous tendencies to fulfill the request embedded in a social interaction since no explicit instructions are imparted to the participants. For example, simply showing an actor moving her arm as if she intended to pour coffee into a cup located close to the observer produces an under-threshold activation of muscles that would be involved in a complementary action (e.g., lifting the cup). These results confirm that action observation does not inevitably lead to an imitative kind of motor facilitation but differs depending on the action context—when the context calls for a complementary action, the excitability pattern reflects the motor preparation of an appropriate response [40,41].

In a first experiment we used eye-tracking procedures to measure the natural allocation of overt spatial attention during passive observation of video clips showing social requests toward the onlooker. In a second eye-tracking experiment we superimposed attentional-capturing dots within the scenes to manipulate the allocation of overt spatial attention. Then, we measured whether diverting attention from the salient parts of the scenes affects motor preparation while TMS was delivered over M1 and motor evoked potentials (MEPs) were recorded in various muscles (Experiment 3).

In terms of direct matching, we hypothesize that CE in the observers’ hand muscles should be compatible with the observed movement, but if and only if attention is directed on the actor’s hand. In terms of complementary actions, we expect a CE increase in the observer’s muscles reflecting a complementary action preparation, which should be almost impermeable to where attention is directed. This is because of the social valence of the stimulus intrinsically requiring less attentional processing.

### Ethical statement

Testing was performed in accordance with the ethics approval by the Institutional Review Board at the University of Padua, in line with the Declaration of Helsinki (Sixth revision, 2008). All participants gave informed written consent before participating in Experiments 1, 2, 3.

## Experiment 1

The aim of this experiment was to ascertain whether overt spatial attention was spontaneously captured by a target object when an observed action implied a social request for it. This preliminary test was conceived as to assess the validity of our stimuli and to provide a solid paradigm to manipulate attentional allocation during the following TMS experiment. For this purpose, eye-tracking procedures were used to measure the spontaneous allocation of overt attention during the observation of interactive and non-interactive action sequences. In particular, we investigated whether overt attention is anchored to the moving hand throughout the action sequence; or whether observers’ overt attention spontaneously shifts on a target object when this is made salient by a request to act upon it.

### Materials and methods

#### Participants

Nineteen right-handed volunteers (8 males and 11 females, age range 21–31 years, mean age 24.8 years) took part in the experiment. All were right-handed according to a Standard Handedness Inventory [42] and had normal or corrected-to-normal vision.

#### Stimuli

Two videoclips (7.2 seconds duration each) were used as experimental stimuli for the ‘Request’ and the ‘No-Request’ conditions, respectively.

**Fig 1:**
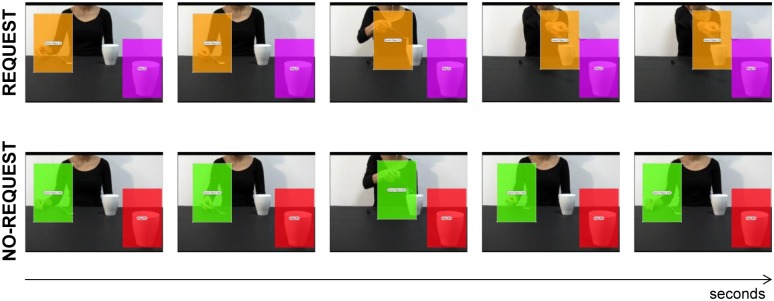
Sequences of events for each experimental condition: ‘Request’ on upper row and ‘No-Request’ on lower row. The overlaid colored rectangular areas represent the AOIs adopted in Experiment 1: the dynamic Hand AOI for the Request (orange) and No-Request (green) conditions, and the static Mug AOI for the Request (violet) and No-Request (red) conditions. Both AOIs had the same dimensions and lasted for the entire duration of the video stimuli.

1. *Request condition*: In this condition the actor grasps a sugar spoon placed on a small starting block, pours sugar into the mug placed next to her on a table and then she stretches out the spoon with some sugar left towards a mug out of her reach, but strategically placed near the observer, thus requiring his/her intervention to lift the mug and complete the complementary action (Fig 1).
2. *No-Request condition*: In this condition the actor grasps the same sugar spoon, pours sugar into the mug placed next to her on a table and then she moves back the spoon with some sugar left to its initial position on the starting block (Fig 1). We edited the video clips in order to hide the model’s head since seeing a face looking at an object causes a rapid, spontaneous shift of spatial attention towards the same target [43,44]. Gaze can act indeed as a confounding factor in biasing the participants’ attentional orientation (for review see [45]).

#### Eye tracking recording

Eye movements were recorded with an infrared T120 Eye Tracker (Tobii Technology, Danderyd, Sweden) set to head-free mode. In this setting, the eye-tracker corrects for head movements and remains very accurate even with changing head position. Eye position was sampled at 120 Hz with a spatial accuracy of 0.5 degrees of visual angle. The eye-tracker calibration was performed at the beginning of the experiment and repeated when necessary by using a standard five-point grid.

#### Procedure

Participants were seated at a distance of 65 cm from the monitor (1280 x 1024 pixels) and they were asked to observe the experimental stimuli (AVI format videos, 25 frames per second) carefully. Each trial started with the presentation of a fixation cross in the center of the screen and participants were instructed to look at the cross for 3 seconds. This ensured that all participants would start observing the video stimuli from the same origin point. Each video clip was only presented once to each participants and randomized across participants. The experimental session lasted approximately ten minutes.

#### Data analysis

**Fig 2:**
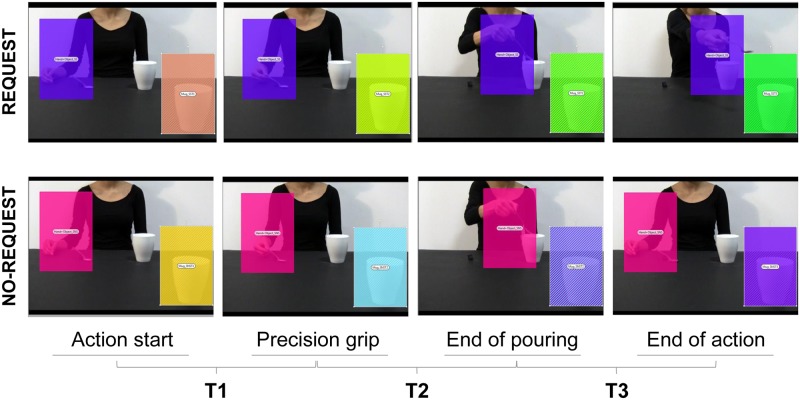
Three time periods adopted to analyze temporal information on Fixation Duration for the Mug AOI. Each column represents a key phase of the action. Eye gaze data in the Mug AOI were segmented into three epochs (T_1_, T_2_, T_3_).

The eye-tracking data were processed by means of the software Tobii Studio 3.1. Areas of Interest (AOIs) were created to investigate fixations targeted to specific regions. A fixation event was computed when gaze remained within 0.5 degree of visual angle for at least 100 ms. For each video two AOIs of the same dimension (217 x 327 pixels) were identified: a) Hand AOI: a dynamic area which included the model’s hand while manipulating the sugar spoon (Fig 1); and b) Mug AOI: a static area involving the mug placed near the observer, in the right corner of the screen (Fig 1). Both AOIs were present for the entire duration of the video stimuli. The total Fixation Duration, namely the average duration in seconds for all fixations within the AOI, was considered for gaze data analysis. A repeated-measure analysis of variance (ANOVA) was conducted on Fixation Duration with condition (Request, No-Request) and AOI (Hand, Mug) as within-subjects factors. A subsequent analysis has been performed in order to investigate the temporal distribution of fixations towards the Mug AOI over time. This experimental design allowed to test whether the adopted social manipulation (i.e., the implicit request to the observer to potentially interact with the mug to fulfil the action) was able to influence gaze deployment over the salient object. Crucially, time course of fixations toward the mug were also measured. Based on the fact that the two action sequences were identical for the first part, a difference in gaze parameters was expected only in the last part of the action, namely when the nearby mug acquired a social valence (Request condition) or not (No-Request condition). To this aim, Fixation Duration has been segmented into three epochs (see Fig 2): i) T_1_, the time elapsing between the start of the action (i.e., hand laying on the table) and the PG on the sugar spoon; ii) T_2_, the time between the PG on the spoon and the end of the action of pouring sugar into the first mug; iii) T_3_, the time between the end of pouring and the final action step (i.e., extending the arm toward the observer for the Request condition and taking back the spoon to the small starting box for the No-Request condition). A repeated-measure ANOVA was then conducted for the Mug AOI on Fixation Duration with condition (Request, No-Request) and time (T_1_, T_2_, T_3_) as within-subjects factors. The Partial Eta Square (η^2^_p_) value was calculated as an estimate of effect size. In the presence of significant interactions, Bonferroni-corrected pairwise comparisons were performed. A significance threshold level of *p* < .05 was set for all statistical analysis, carried out with SPSS software package (SPSS Inc, Chicago, IL, USA).

### Results

#### Spatial pattern of gaze engagement at the salient object

**Fig 3:**
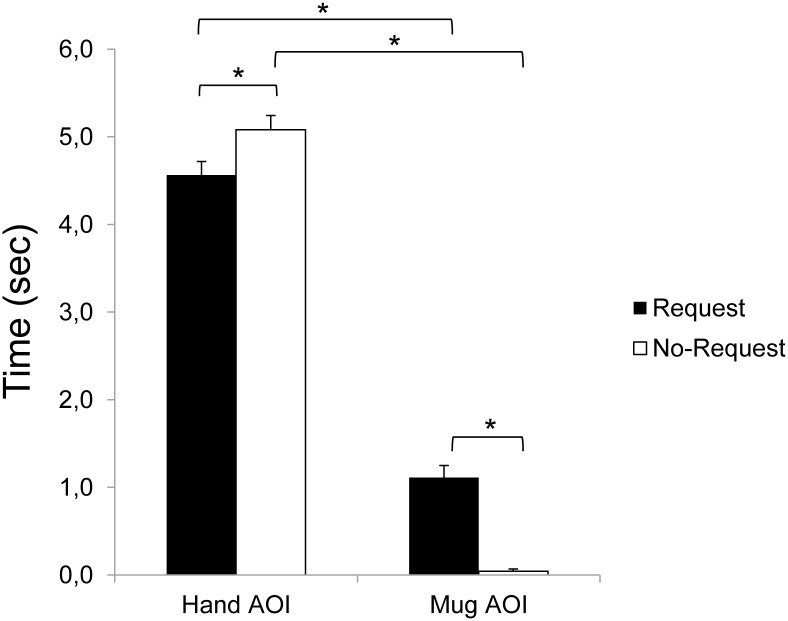
Fixation Duration on the Hand and Mug AOIs for the Request and No-Request conditions. Results show a statistically significant longer Fixation Duration for the Mug AOI for the Request condition with respect to the No-Request condition. Bars indicate the standard error of the means. Asterisks indicate statistically significant comparisons (*p*_*s*_ < 0.05).

The ANOVA on the mean Fixation Duration (i.e., the time spent fixating within the AOI for each video clip) showed significant main effects for both condition (F_1,18_ = 7.29, *p* = 0.015, η^2^_p_ = 0.29) and AOI (F_1,18_ = 550.75, *p* < 0.001, η^2^_p_ = 0.97), and a significant interaction of condition by AOI (F_1,18_ = 33.11, *p* < 0.001, η^2^_p_ = 0.65). The results obtained from the post-hoc contrasts exploring the interaction showed significantly longer fixation times for the Hand AOI compared to the Mug AOI for both the Request and the No-Request conditions (*p*_*s*_ < 0.001). This result is in accordance with the salience of the observed moving hand in attracting overt attention during the observation of the two types of actions. Statistically significant longer Fixation Duration for the Mug AOI for the Request condition with respect to the No-Request condition (*p* < 0.001) was found. Participants looked longer the mug placed next to them when a social request was embedded in the action. Accordingly, participants fixate for a shorter time the Hand AOI in the Request condition compared to the No-Request condition (*p* = 0.019). Results are graphically summarized in Fig 3.

#### Temporal pattern of gaze engagement directed to the salient object

**Fig 4:**
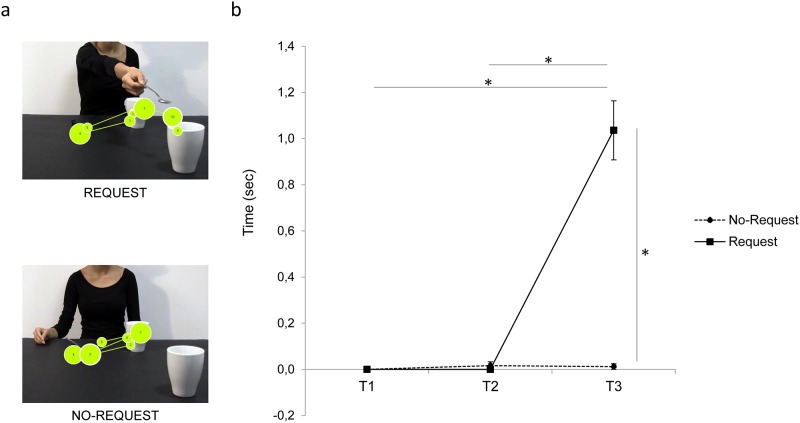
Fixation Duration on the Mug AOI for the Request and the No-Request conditions. Panel a) shows an example of gaze plots (i.e., the representation of the sequence of eye movements, displaying order and duration of fixations) for the Request and the No-Request conditions from a representative participant. The lines represent saccades, while the circles represent gaze fixations and circle areas are proportional to fixation lengths. Results in panel b) show longer Fixation Duration at T_3_ when the hand is stretched toward the out-of-reach mug (Request condition) compared to the earlier phases of the action. Bars indicates standard error of the means. Asterisks indicate statistically significant comparisons (*p*_*s*_ < 0.001).

The present analysis aimed at measuring the temporal aspects of gaze when directed towards the target object (i.e., the mug on the right corner of the screen) during the observation of interactive and non-interactive action sequences. The ANOVA on the mean Fixation duration showed a significant main effect of both condition (F_1,18_ = 62.73, *p* < 0.001, η^2^_p_ = 0.78) and time (F_2,36_ = 67.47, *p* < 0.001, η^2^_p_ = 0.79), and a significant interaction of condition by time (F_2,36_ = 60.66, *p* < 0.001, η^2^_p_ = 0.77). The results obtained from the post-hoc contrasts exploring the interaction showed significantly longer fixation time for the Request condition at the T_3_ time period compared to either the T_1_ and the T_2_ time periods (*p*_*s*_ < 0.001). Furthermore, the results show a significant longer fixation time at T_3_ for the Request compared to the No-Request condition (*p* < 0.001). Overall, participants fixate longer the Mug AOI when the implicit request to interact is unfolded (i.e., at T_3_, when the hand is stretched toward the out-of-reach mug) compared to the earlier phases of the action. Crucially, eye gaze is spontaneously shifted toward the mug (i.e., the object with which interact to complete the complementary request) only during the final part of the interactive condition. Results for Fixation Duration on Mug AOI in T_1_, T_2_ and T_3_ are graphically summarized in Fig 4b. In addition, Fig 4a shows the spatial and temporal distribution of fixations over time (gaze plot) for both the Request and No-Request conditions for a representative participant.

## Experiment 2

Experiment two aimed at determining eye gaze modifications when exogenous attention toward specific parts of the visual scene was manipulated by means of a red-dot. Notably, attention can be shifted toward specific parts of the visual scene either voluntarily, also referred as to endogenous attention, or automatically, which is referred as to exogenous (or stimulus-driven) attention [46]. Exogenous visual attention is captured by salient physical properties of the visual stimuli like motion [22] and social salience [38]. Moreover, attention orienting can be overtly or covertly allocated toward a stimulus [47]. In the first case eye movements are directed toward it (to bring the stimulus at the fovea, where visual acuity reaches its peak), whereas cover orienting occurs independently from eye movements. Here, by means of eye-tracking procedures, we investigated whether the appearance of a short-lasting dot superimposed on the video clips was able to capture overt attention despite the salience of the observed biological movement and of the target object. It is possible, however, that participants were able to detect the dot presence by simply directing their covert attention towards it. In a follow-up test we therefore investigated whether the dot was correctly detected by the participants regardless of gaze orienting.

### Methods

The method in Experiment 2 was identical to Experiment 1 with the following exceptions regarding the experimental sample, the type of stimuli and the data analysis process.

#### Participants

Twenty-one different right handed participants (9 male, 12 female) with mean age of 25.05 (SD = 6.35) took part in the experiment.

#### Stimuli

**Fig 5:**
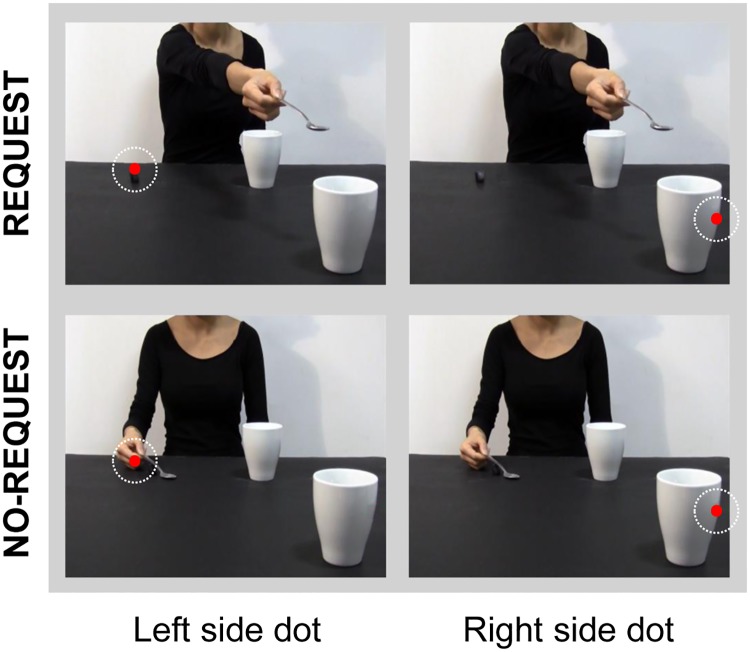
Experimental stimuli adopted for Experiment 2. An attentional-capturing red dot was briefly presented in either one of two specific positions: ‘left side’ (see left column), over the starting block for the Request condition and over the actress’s hand moving back to the initial position for the No-Request condition; and ‘right side’ (see right column), over the out-of-reach mug located on the right side of the screen (Request and No-Request conditions). White dotted circles indicate the red dot position.

The same videoclips as for the Experiment 1 were adopted. Crucially, a red dot (note that from now on it would be regarded as ‘dot’) was superimposed on the videos and briefly presented in order to elicit the shift of the observer’s exogenous attention to different locations in the visual scene. The dot (40 x 40 pixels, 120 ms duration) was presented at the end of both action sequences (4460 ms from video onset), in either one of two specific locations: (i) *‘left side’* (Fig 5, left column), over the actor’s hand moving back to the initial position on the left side of the screen (No-Request condition) or on the same spatial location over the starting box (Request condition); and (ii) *‘right side’* (Fig 5, right column), over the out-of-reach mug located on the right side of the screen (for both the Request and the No-Request conditions). In particular, the location of the ‘right side’ cue was selected on the basis of the findings obtained in Experiment 1, showing that for the Request video the Mug AOI was significantly more attended by the observers during the last part of the action sequence than for the No-Request video. Each video lasted 5540 ms and was preceded by the presentation of a white fixation cross on a black background for 3000 ms to ensure that participants would start the observation from a neutral and fixed position.

#### Procedure

The same procedures as for Experiment 1 were adopted. Each video was presented five times in a random order. The experimental session lasted approximately fifteen minutes. At the end of the experiment a *follow-up* test was performed to verify whether the dot was covertly detected by the participants. Participants were asked to report whether they saw the dot or not at the end of each video presentation. The dot was inserted in the 80% of trials. Participants’ performance was assessed by means of proportion of correct responses (accuracy).

#### Data analysis

**Fig 6:**
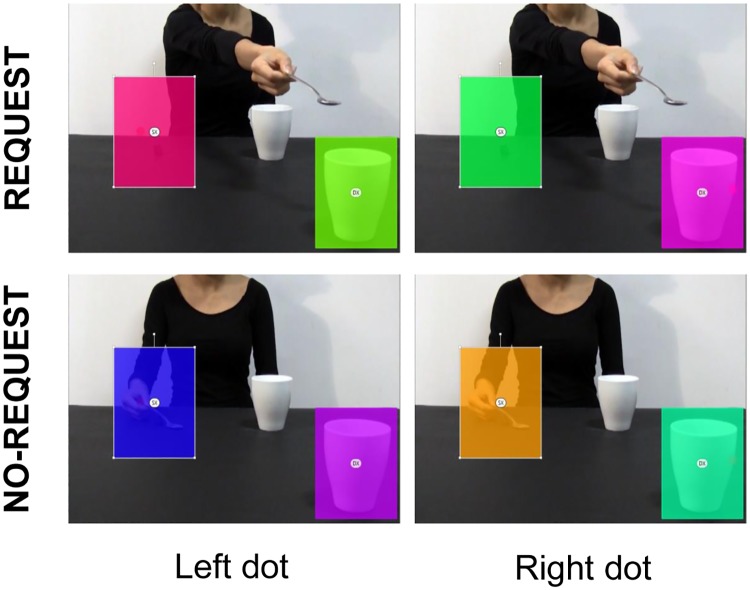
Overlaid colored rectangular areas represent the AOIs adopted for Experiment 2. ‘Left AOI’ includes the area in which the left dot appears (see the rectangle on the left on each figure); ‘Right AOI’ includes the area in which the right dot appears (see right-side rectangle on each figure).

As for Experiment 1, eye gaze data were analyzed by means of the software Tobii Studio 3.2. Eight static Areas of Interest (AOIs) with the same dimension (188 x 237 pixels) were defined to investigate gaze fixations targeted to the areas of the visual scenes in which the dot was presented. Specifically, two AOIs were created for each video: a) ‘Left AOI’, including the area in which the left dot was presented (see Fig 6, left column), and b) ‘Right AOI’, including the area in which the right dot was showed (see Fig 6, right column). The analysis of eye gaze for each video presentation was carried out in a time window that started with the dot’s appearance and ended after 320 ms (dot presentation duration: 120 ms). This temporal window was adopted not to include in the analysis the Inhibition of Return (IOR) phenomenon (i.e., the inhibition of re-orienting attention to a previously explored location) [48,49]. In order to detect the orienting of attention to a specific location induced by the brief presentation of an exogenous attention-capturing dot, Fixation Count (the number of fixations within the AOI) was considered. In fact, given the short time window adopted, the total Fixation Duration (the average duration for all fixations within the AOI) would not represent the most sensitive parameter to use. A repeated-measure ANOVA was conducted on Fixation Count with condition (Request, No-Request), dot location (Left side, Right side) and AOI (Left, Right) as within-subjects factors. The Partial Eta Square (η^2^_p_) value was calculated as an estimate of effect size. In presence of significant interactions, post-hoc comparisons were performed using the Bonferroni correction. Significance threshold was set at *p* < 0.05 for all statistical analysis carried out with SPSS software package (SPSS Inc., Chicago, IL, USA). As concern the *follow-up* test, accuracy values were submitted to a repeated-measure ANOVA with condition (Request, No-Request), dot location (Left side, Right side, no dot) as within-subjects factors. Bonferroni corrections were applied (alpha level 0.05).

### Results

The ANOVA on the Fixation Count showed a significant main effect of condition (F_1,20_ = 73.62, *p* < 0.001, η^2^_p_ = 0.79), AOI (F_1,20_ = 132.65, *p* < 0.001, η^2^_p_ = 0.87), a significant two-way interaction of condition by AOI (F_1,20_ = 79.31, *p* < 0.001, η^2^_p_ = 0.80) and of dot location by AOI (F_1,20_ = 22.88, *p* = 0.008, η^2^_p_ = 0.30) and a three-way interaction of condition by dot location by AOI (F_1,20_ = 5.71, *p* = 0.027, η^2^_p_ = 0.22). The results obtained from the post-hoc contrasts exploring the source of the significant three-way interaction are outlined as follows.

#### Request condition: Dots attracts eye gaze

**Fig 7:**
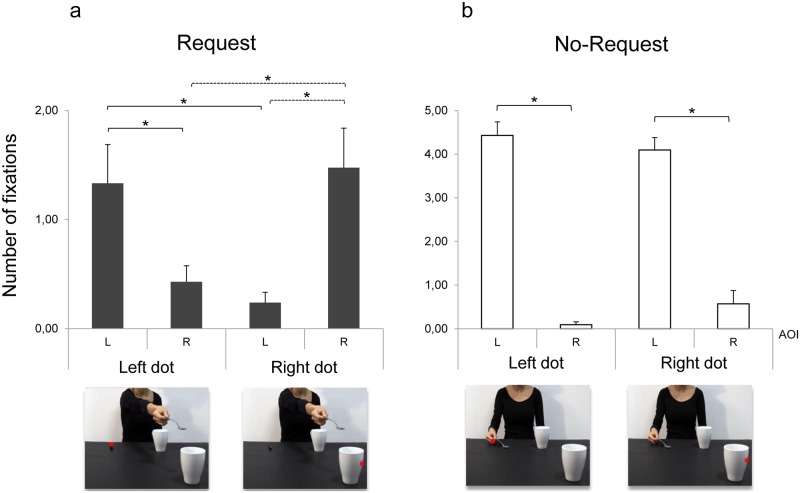
Fixation Count for Request and No-Request conditions in Experiment 2. Left and Right AOIs (‘L’ and ‘R’, respectively; y-axis) and dot location (‘left dot’ and ‘right dot’; y-axis) are represented. Results show that dot manipulation is effective in attracting the observers’ gaze for the Request but not for the No-Request condition. Error bars indicates standard error of the means. Asterisks indicate statistically significant comparisons (*p* < 0.05).

For the Request condition, presenting the dot on the left side (i.e., over the small starting box) increased Fixation Count for the Left AOI compared to the Right AOI (*p* = 0.033; Fig 7a). Fixation Count for the Left AOI when the dot appeared on the left side was also higher than Fixation Count for the same AOI when the dot appeared on the right side (*p* = 0.005; Fig 7a). Similarly, Fixation Count in the Right AOI was higher when the dot appeared on the right compared to the left side (*p* = 0.005) and compared to the same AOI when the dot was instead presented on the left side (*p* = 0.005; Fig 7a). To summarize, in the interactive context the dot manipulation was able to attract eye gaze toward the area of presentation.

#### No-Request condition: Eye gaze is anchored on biological movement

Results for the No-Request condition showed that Fixation Count was higher in the Left AOI compared to the Right AOI regardless of dot location (*p*_*s*_ < 0.001; Fig 7b). Although participants tended to fixate more frequently the Right compared to the Left AOI when the dot appeared on the right side (*p* = 0.11; Fig 7b), eye gaze remained anchored on the actor’s hand.

#### Dots attract covert attention

Overall accuracy for the *follow-up* test was 99%. The ANOVA on the proportion of correct responses yielded no statistically significant main effects (*p*_*s*_ > 0.05) nor interactions (*p*_*s*_ > 0.05), meaning that participants correctly reported the presence of the dot irrespective of condition and dot location. Notably, this allows to conclude that the ‘dot’ manipulation was effective in attracting participants’ covert attention even when eye gaze remained anchored on the actor’s hand in the No-Request condition.

## Experiment 3

To summarize, results from Experiment 1 showed that attention was spontaneously captured by the out-of-reach object when the observed action implied a social request. Experiment 2 indicated that gaze direction can be manipulated by means of attentional-capturing dots, except at the end of the No-Request action: in that case, fixations were targeted to the actor’s hand. In the present Experiment, single-pulse TMS (spTMS) was delivered on M1 to probe the influence of overt spatial attention on the participants’ corticospinal excitability during observation of social and non-social actions.

### Materials and methods

#### Participants

Thirty volunteers (8 males and 22 females, age range 20–28 years, mean age 23 years) took part in the experiment. All were right-handed according to a Standard Handedness Inventory [42] and had normal or corrected-to-normal vision. They were free from any contraindication to TMS [50] and none of them experienced discomfort during the experiment. All participants were naïve as to the purpose of the study. At the end of the experimental session detailed information concerning the study was provided. Participants were financially compensated for their time.

#### Stimuli

The same stimuli as for Experiment 2 were adopted (see Fig 6), namely the Request and the No-Request action sequences in which attention-capturing dots were superimposed on the actions in either one of two specific locations (i.e., ‘left side’, ‘right side’). Crucially, in both video clips the model grasps the sugar spoon with her right hand using a precision grip (PG), whilst the mug requires the use of a whole hand grasp (WHG) in order to be handled. So, the type of grasp observed (i.e., a PG) and the one that is required to interact in the Request context (i.e., a WHG) are reciprocally mismatched. It follows that dots positioned on the left side were always associated with precision grips (Fig 6, left column), whereas Right side dots, positioned on the mug, were associated with a WHG (Fig 6, right column). This experimental design allowed us to disentangle different types of motor preparations and to control for object-related motor affordances [51,52]. Each video lasted 5540 ms and the animation effect was obtained by presenting a series of single frames each lasting 30 ms (resolution 1920 x 1080 pixels, color depth 32 bits) following the first frame lasting 800 ms.

#### Procedure

Participants were tested individually in a single experimental session lasting approximately one hour. They sat comfortably in an armchair, keeping their arms fully relaxed in a natural position, with their hands pronated and resting on a pillow. They passively observed the video clips that were presented on a 24” monitor (resolution 1920 x 1080 pixels, refresh frequency 120 Hz) set at eye level (the eye-screen distance was 80 cm). Notably, participants were not instructed to voluntary focus on the actor’s movements not to induce different attentional focuses. In order to ensure attention to the video clips, they were told that at the end of the experiment they would be questioned about the stimuli presented. TMS-induced motor evoked potentials (MEPs) were acquired from the participants’ right first dorsal interosseous (FDI) and adbuctor digiti minimi (ADM) muscles of the right hand. These muscles were chosen because of their differential activation during the execution of PG versus WHG (i.e., a higher activation of FDI for PG and of ADM for WHG). As demonstrated in a series of previous studies [41,53,54], seeing an actor in a frontal position signaling a request near a salient object strategically placed out of her reach induces a modulation in the observer’s MEP amplitudes that is consistent with the intention to accept the request (here, reaching for and grasping the mug with a WHG) rather than with the tendency to resonate with the observed action (here, performing a PG). Notably, placing the object in the observer’s peripersonal space is a crucial factor for inducing a function-related affordance [55–58]. A single TMS pulse was released during each video presentation at 5750 ms, after 150 ms from the dot’s presentation over the ‘left side’ or ‘right side’ position on the scene. This timing was chosen based on previous literature showing that visuomotor mapping corresponding to the observed motor act occurs at around 150 ms [59]. Since the two video-clips only differed in the final phase of the action sequence, at 5600 ms, and dot presentation was synchronized with this timing, the degree to which the motor system was activated during spTMS provided an index of the CS activity elicited by action observation and modulated by dot presentation. We synchronized the timing of TMS stimulation with the occurrence of dot presentation to avoid that the general pattern of attention could be restored right after dot presentation. We are confident that the modulation we will measure is genuinely due to an automatic shift of attention (as documented in Experiment 1) rather than to a general effect of attentional salience of the two stimuli. Indeed, previous experiments with the very same video clips but no dot presentation did not report such results [40,60]. The order of the videoclips was randomized across participants. A total of 80 MEPs (2 muscles x 2 conditions x 2 dot positions x 10 repetitions) was recorded for each participant. Prior and after the experimental block, each participant’s baseline CE was assessed by acquiring 10 MEP while they passively watched on the computer screen a white-colored fixation cross on a black background. Possible variations in CE related to TMS per se were assessed by comparing the MEP amplitudes recorded during the two baseline periods (20 MEPs in total). Their average amplitude was then utilized to set each participant’s individual baseline for data normalization procedures. An inter-pulse interval lasting 10 s was presented between trials in order to avoid any short-term conditioning effect [61]. During the resting period, a message reminding the participants to remain fully relaxed appeared on the screen for the first 5 seconds, and a fixation cross was presented for the remaining 5 seconds. The cross presentation ensured participants to start observing the videos from a neutral gaze position in each trial. Stimuli presentation, timing of TMS stimulation and EMG recordings were managed by E-Prime V2.0 software (Psychology Software Tools Inc., Pittsburgh, PA, USA) running on a PC.

#### Transcranial magnetic stimulation and electromyography recording

Single-pulse TMS was administered via a standard eight-shaped focal coil connected to a monophasic Magstim 200 stimulator (Magstim Co., Whitland, UK). The coil was placed tangentially over the left primary motor cortex (M1) contralateral to the examined muscles, with the handle pointing caudally and laterally about 45° from the midline [62,63]. The optimal scalp position (OSP; i.e., the location on the scalp eliciting MEPs simultaneously from the FDI and ADM muscles with the minimum stimulation intensity) was marked on a tight-fitting cap worn by the participant in order to allow the same coil positioning during the entire study. The individual resting motor threshold (rMT; i.e., the lowest stimulus intensity at which TMS generate MEPs of at least 50 μV in relaxed muscles in 5 out of 10 consecutive pulses) was determined [64]. The stimulation intensity was then set at 120% of the rMT to record a clear and stable EMG signal throughout the experiment. rMT ranged from 34 to 61% (mean = 47%, SD = 7) of the maximum stimulator output. During the experimental sessions the coil was held by a tripod and continuously checked by the experimenters to maintain a constant positioning with respect to the marked OSP. Surface Ag/AgCl electrodes (1 cm diameter) were positioned over the FDI and ADM muscles in a belly-tendon montage, with the active electrode on the muscle belly and the reference over the interphalangeal joint. The ground electrode was positioned over the participant’s left wrist. Skin impedance, evaluated at rest prior to beginning the experimental session, was considered of good quality when below the threshold level (5 Ohm). Electrodes were connected to an isolable portable ExG input box linked to the main EMG amplifier for signal transmission via a twin fiber optic cable (Professional BrainAmp ExG MR, Munich, Germany). The raw myographic signals were band-pass filtered (20 Hz-1 kHz), amplified prior to being digitalized (5 KHz sampling rate), and stored on a computer for off-line analysis. Trials in which any peak-to-peak EMG activity greater than 50 μV was present in the 100 ms window preceding the TMS pulse were discarded to prevent contamination of MEP measurements by background EMG activity. EMG data were collected for 300 ms after the TMS pulse.

#### Data analysis

Individual peak-to-peak MEP amplitude (mV) was calculated offline and averaged for each participant and experimental condition using Brain Vision Analyzer software (Brain Products GmbH, Munich, Germany). MEP amplitudes deviating more than 3 SD from the mean for each subject and trials contaminated by muscular pre-activation were excluded as outliers (< 5%). A paired sample *t*-test (two-tailed) was used to compare the amplitude of MEPs recorded during the two baseline periods carried out at the beginning and at the end of each block. Mean peak-to-peak MEP amplitudes were then normalized with respect to the basal MEP measured at rest (i.e., individual mean MEP amplitude recorded during the two baseline periods) as follows: MEP ratio = MEPobtained / MEPbaseline. A repeated-measures analysis of variance (ANOVA) was conducted on the MEP ratios with condition (Request, No-Request), dot position (left side, right side) and muscle (FDI, ADM) as within-subjects factors. The Partial Eta Square (η^2^_p_) value was calculated as an estimate of effect size. In the presence of significant interactions, Bonferroni-corrected pairwise comparisons were performed. A significance threshold level of *p* < .05 was set for all statistical analysis, carried out with SPSS software package (SPSS Inc, Chicago, IL, USA).

### Results

The mean raw MEP amplitudes recorded at the beginning and at the end of the experimental session were not significantly different for either the FDI (t_29_ = -.287, *p* = .783) or the ADM (t_29_ = .638, *p* = .529) muscles. Therefore, TMS *per se* did not induce any nonspecific change in CE that could have biased the results. The ANOVA on normalized MEP amplitudes showed a significant muscle by condition interaction (F_1,29_ = 7.350, *p* = .011, η^2^_p_ = .202) and a 3-way interaction of muscle by condition by dot position (F_1,29_ = 7.436, *p* = .011, η^2^_p_ = .204). The results obtained from the post-hoc contrasts exploring the significant 3-way interaction are outlined as follows.

#### Attentional interference modulates direct matching

**Fig 8:**
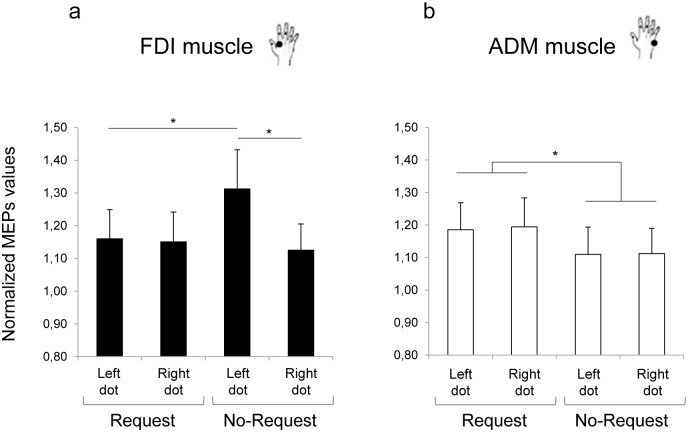
Normalized mean MEP amplitudes for Request and No-Request conditions in Experiment 3. MEPs were recorded from: a) first dorsal interosseous (FDI) and b) abductor digiti minimi (ADM) muscles. Results show that direct matching depend on attentional allocation, whereas diverting attention do not modulate the preparation of a complementary response. Error bars indicate standard error of the means. Asterisks indicate statistically significant comparisons (*p* < 0.05).

As concerns the No-Request condition (Fig 8), a motor facilitation effect was shown when the dot was presented on the actor’s hand (i.e., Left dot). MEP amplitude of observers’ FDI muscle (i.e., the muscle involved in precision grip) was significantly greater than in the Request condition, though the dot was located on the same location (i.e., left side; *p* = .030, Fig 8a). Direct matching was reduced when the dot was located on the out-of-reach mug: FDI muscle activity was decreased in the Right with respect to the Left dot condition (*p* = .010, Fig 8a). As suggested by previous literature, direct matching seems to depend on attentional allocation directed to body parts [24, 25].

#### Diverting attention do NOT affect preparation for a complementary response

ADM muscle activity was statistically higher for the Request than for the no-Request condition (1.19 vs. 1.11, respectively; t_29_ = 2.15, *p* = .035). In the Request condition, in particular, ADM muscle was equally activated when the dot was positioned on the out-of-reach mug and when it was positioned on the left side of the screen (*p* = .856; Fig 8b). Diverting attention did not modulate the preparation of a complementary response.

## Discussion

The current study aimed at clarifying the influence of attentional interference during action observation from both a behavioral and a neurophysiological perspective. Our data show that during action observation, corticospinal excitability in the relevant muscles is reduced by the brief appearance of a flashing dot in the contralateral space. Conversely, the appearance of the dot does not impair motor preparation for a complementary response.

Here we offer evidence that the mechanisms underlying action observations are more complex and multifaceted than previously thought.

### Overt attention and direct matching

Our data confirm and extend previous behavioral [24,25], neuroimaging [26–28] and neurophysiological [29,30] results suggesting that motor system activation during action observation can be automatic, but some attentional mechanisms play a crucial role. Here we found a selective increase of the observers’ corticospinal excitability for the FDI muscle only when the dot was presented on the hand in the No-Request condition. Notably, FDI muscle is specifically involved in both the execution [65] and observation [66] of precision grips. The short-term appearance of a dot in the contralateral space with respect to the moving hand significantly diminished the matching muscular activation. Such decrease makes functional sense in terms of selective filtering of task-irrelevant stimuli and would support recent evidence showing specific reduction of neural activity under conditions of high attentional load for biological movement [26].

A plausible alternative explanation might be that the smaller CE observed in FDI muscle when the dot was placed in the contralateral side relates to object affordance [51,52]. This is because the distracting dot for the No-Request video appeared on the out-of-reach mug thus drawing attention to it. In turn, paying attention to the mug might have triggered the motor plan to grasp it (i.e., WHG). If this were the case, then we should have found an increased ADM activation at the expense of FDI muscle. However, no statistically significant activation was found in the little finger muscle in this condition. Therefore we feel that the attentional load hypothesis appears to be more likely.

### Diverting attention do NOT affect complementary responses

A CE modulation compatible with a complementary response on the target object (i.e., a WHG) was shown in the observers’ muscles when the observed action was calling for a joint intervention (i.e., Request condition). More importantly, this activation was not affected when the dot was diverting overt attention to the contralateral side of the scene, with respect to the object. It seems therefore that social motor preparation is resistant to modulation by top-down mechanisms such as visuospatial attention. This would be analogous to the processing of biologically relevant stimuli (e.g., threatening facial expressions), which elicit neural activity despite participants’ attention is directed toward a distractor stimulus [67,68]. The findings outlined here are in line with previous literature suggesting that engaging in complementary actions is made possible by immediate apprehension of another person’s intentions towards a salient object [69–71] and its affordances.

In this regard, *complementary affordances* refer to all those possibilities for interaction provided by others, which activate appropriate motor programs aiming to bring a common goal to completion [41]. We directly perceive and selectively respond to complementary actions, even in situations in which such involvement does not take place. The activation of a complementary affordance is extremely powerful, and present data suggests that the automatic decoding of others’ actions influences our behavior beyond attentional involvement, maximizing the efficiency of our socially appropriate responses [72,73]. In future studies, the adoption of a double dissociation control showing WHG grasping movements and an object triggering a complementary pincer grip action (see [40,54,60] for an example) would permit to ascertain that the effects documented here do not reflect different sensibility to attention of the cortical representation of the two muscles.

Moreover, the present study support and extend previous data showing that when gaze cues (i.e., the primary source of information allowing for the prediction of other’s action goals) are not available, participants orient their attention to the others’ actions as a secondary source of information [74,75]. Notably, the deployment of visuospatial attention and the programming of saccades are governed by the inferred likelihood of events [76]. People shift their attention towards what they *expect* other people will look at [77,78]. These prediction biases can lead to similar attentional shifts as directly perceived gaze [79]. In our study it is conceivable that prediction of others’ behavior might have anchored the observer’s covert attention on the salient object (see Follow-up study), regardless of cue manipulation. This is in line with the predictive coding theory [80] stating that we calculate the consequences of an observed movement through auto-generated forward models [81,82].

It could be argued that this effect is due to the presence of a biological movement close to the object, without the effect being intrinsically social. If this were the case, then a simple arrow cue pointing towards the object would produce similar findings. However, results from previous studies in which the social request was substituted by an arrow did not provided support for this view [53,83]. Rather, we suggest that the motor system is preferentially tuned to meaningful actions of interactive partners.

### Attention: Serial or parallel coding?

Several studies have suggested that attention can be distributed and observers can select sensory information independently from separate location [84–88], but only when the display does not contain novel onset distractor stimuli, which automatically capture attention [89]. More recently, Eimer and Grubert [90] challenged this view demonstrating that observers can also allocate focal attention in parallel to two different target objects appearing in rapid succession at different locations. In other words, attention can be maintained at its previous location while it is simultaneously allocated to a new target object. However, most research on divided spatial attention has only made use of artificial experimental stimuli, such as simple geometrical shapes. How we divide attentional resources in more complex, social contexts remains largely unexplored. In this respect, Kourtis and colleagues [91] clearly demonstrated that when planning to engage in a joint action, people can covertly distribute their attention between self-relevant and other-relevant locations. This evidence stems from a situation in which interacting agents performed the same movement (i.e., clinking two glasses together). When considering, however, observation of movements that require incongruent rather than imitative actions (i.e., complementary actions) [41,92], no evidence on attention deployment was still available.

In the present study we showed that presentation of a distractor cue—able to capture eye gaze—did not hinder the motor preparation of a social response. To date, our study offers the first direct assessment that overt orienting of spatial attention and corticospinal excitability during action observation are unrelated. This is in line with recent neurophysiological evidence showing that classical markers of action observation such as motor priming and interference are unrelated with respect to motor cortex activations and cannot replace more reliable measures of the action-perception system [93]. It seems possible, then, that a parallel allocation of attention allowed participants to attend both the salient object and the cue appearing in the contralateral side without interference in terms of CE. Evidence suggests that stimuli with a social valence require less attention to be processed [38,67]. A low processing load would allow attentional resources to ‘spill over’ to the processing of other irrelevant features of a stimulus [94,95]. Notably, visual search in composite natural situations can be remarkably fast and efficient despite the overwhelming amount of information [96,97]. Fagioli & Macaluso [98] recently suggested that two factors may influence attentional control in these contexts. First, real-world objects are recognized more quickly when they are familiar object, a phenomenon termed “ultra-rapid categorization” [99]. They are thus categorized in a pre-attentive manner, with little requirements of top-down control [100]. Second, visual search and recognition are influenced by prior knowledge about the spatial arrangement of objects within natural scenes (‘contextual cueing effect’) [101]. In the present experiment, the contribution of these factors may have led to an easier and more efficient processing of the salient object by the observer, but just when the action was calling for a social interaction.

## Conclusion

The present research suggests a double dissociation between overt attentional allocation, neurophysiological mapping (i.e., direct matching), and social motor preparation (i.e., the activation of an appropriate response to the observed action). This is a novel and interesting finding and it is consistent with recent evidence showing that behavioral and TMS markers of action observation might reflect distinct neuronal processes [93].

The nature of the link between perception and action continues to be debated: is it learning how to interact with other people sub-served by general stimulus-response (S-R) associations, or is it treated in a special way? Understanding the role played by overt visuospatial attention in social interactive contexts might prove to be crucial to get to the core of the matter. While further research is needed to determine the specific time-course of the attentional modulation during social interactions and the interplay between overt and covert attention, our results are among the first to provide evidence that social motor preparation is impervious to attentional interference.
